# Data supporting the *in situ* synthesis by organometallic method of Vulcan supported PdNi nanostructures for hydrogen evolution reaction in alkaline solution

**DOI:** 10.1016/j.dib.2022.108256

**Published:** 2022-05-10

**Authors:** J.J. de la Cruz-Cruz, M.A. Domínguez-Crespo, E. Ramírez-Meneses, A.M. Torres-Huerta, S.B. Brachetti-Sibaja, A.E. Rodríguez-Salazar, E. Pastor, L.E. González-Sánchez

**Affiliations:** a Instituto Politécnico Nacional, Centro de Investigación en Ciencia Aplicada y Tecnología Avanzada, CICATA, Unidad Altamira, Carretera Tampico-Puerto Industrial, km 14.5, Altamira, Tamaulipas, CP 89600, México; b Instituto Politécnico Nacional, UPIIH, Ciudad del conocimiento y la cultura. Carretera Pachuca-Actopan km. 1+500 San Agustín Tlaxiaca, Hidalgo, México; c Departamento de Ingeniería Química, Industrial y de Alimentos, Universidad Iberoamericana, Prolongación Paseo de la Reforma 880, Lomas de Santa Fe. C.P. 01219, Ciudad de México, México; d TecNM, Instituto Tecnológico de Cd. Madero. Ave. Primero de Mayo s/n Col. Los Mangos, C.P. 89440, Cd. Madero Tam., México; e Instituto Politécnico Nacional, Centro de Investigación en Ciencia Aplicada y Tecnología Avanzada Unidad-Querétaro, Cerro Blanco No. 141 Col. Colinas del Cimatario, C.P. 76090. Querétaro, Qro. México; f Instituto de Materiales y Nanotecnología, Departamento de Química, Universidad de La Laguna, A. 456, La Laguna, Santa Cruz de Tenerife Apartado Postal 38200, España

**Keywords:** Fuel cells, Methanol oxidation reaction, alkaline media, Pt base catalysts, organometallic method

## Abstract

This document presents the supporting information for the evaluation of the role of Ni amount during the *in situ* synthesis of vulcan supported PdNi nanostructures using an organometallic approach for hydrogen evolution reaction in alkaline medium [1]. The data here presented included analysis of deconvolution during structural characterization, chemical composition and transmission electron microscopy. The information also contains complement data of cyclic voltammograms during activation in alkaline media. Supplement data of electrochemical impedance spectroscopy measurements at two different overpotentials (-100 and -300 mV) and temperatures on the onset potential for hydrogen evolution reaction (HER) are also showed in this paper. The files can be used as a reference to determinate the effect of adding different in situ amount of Ni to Pd/C catalysts in presence of 2 equivalents of hexadecylamine (HDA) in order to improve the electrochemical performance on HER using an adjusted organometallic method. The data provided in this article have not been previously published and are available to enable critical or extended analyses.

## Specifications Table

**Table:** 

Subject	Materials science
Specific subject area	Characterization of electrode materials for their application in low temperature fuel cells
Type of data	Tables Figures
How data were acquired	X-ray photoelectron spectroscopy (XPS, PHI-548 equipment), X-ray diffraction (XRD, Brucker D8 Advanced), Transmission electron microscopy (JEM-ARM200CF, JEOL operating at 200 kV). Electrochemical measurements: cyclic voltammetry and electrochemical impedance spectroscopy were performed in a pontentiostat/galvanostat (AUTOLAB Metrohm, 50404).
Data format	Raw, filtered, fitted curves and analyzed data.
Description of data collection	The chemical and microstructural data were acquired on synthesized samples containing different amounts of Ni (30, 50 and 70 wt.%) in Pd catalysts. The electrochemical data were obtained using 50^th^ potential cycles for stabilization, and 50^th^ cycles in 1M KOH at scan rate of 10 mV s^−1^. The EIS measurements were acquired at two different overpotentials (-100 and -300 mV) and two temperatures (30 and 50°C) in the hydrogen evolution region from open circuit potential with respect to Hg/HgSO_4_ electrode. The relationship between chemical, structural morphological and electrochemical performance of Pd_x_Ni_1-x_ electrode materials. The data were acquired in the as-prepared samples without special treatment.
Data source location	XPS spectra were taken at Centro de Nanociencias y Nanotecnología, Carr. Tijuana-Ensenada km 107, Playitas, C.P.22860, Ensenada B.C., México TEM images were taken at the Centro de Nanociencias micro y Nanotecnologías del Instituto Politécnico Nacional C.P. 07300 Ciudad de México, México. CV, EIS techniques and XRD patterns were collected at Centro de investigación en Ciencia Aplicada y Tecnología Avanzada del Instituto Politécnico Nacional, C.P. 89600 Altamira, Tamaulipas, México.
Data accessibility	Repository name: Mendeley Data Data identification number: 10.17632/www8pfwjm8.2 Direct link to the dataset: https://data.mendeley.com/datasets/www8pfwjm8/2
Related research article	J. J. De la Cruz-Cruz, M. A. Domínguez-Crespo, E. Ramírez-Meneses, A. M. Torres-Huerta, S. B. Brachetti-Sibaja, A. E. Rodríguez-Salazar, E. Pastor, L. E. González-Sánchez*. In situ* synthesis by organometallic method of Vulcan supported PdNi nanostructures for Hydrogen evolution reaction in alkaline solution. Int. J. Hydrog. Energy. https://doi.org/10.1016/j.ijhydene.2022.02.226[1].

## Value of the Data

• The data are useful to verify the formation of PdNi nanostructures and to compare their performance for the hydrogen evolution reaction using different methods.
• The data may be beneficial or used by researchers to compare palladium-based nanostructures with potential applications as electrode materials in PEMFC devices.
• The data are also valuable because compared changes in the crystallite size with different amounts of Ni to Pd/C catalysts when an organometallic approach is used in presence of hexadecylamine (HDA) as stabilizer.
• The data show that the HDA can be eliminated by annealing process without affecting the dispersion on the vulcan carbon, particle size and nominal composition of the Pd_x_Ni_1-x_/C nanostructures using an organometallic approach as synthesis method.
• The data are valuable because can be used to show the effect of the annealing process (300°C) on the dispersion, particle sizes and catalytic activity of the PdNi electrode materials on the HER in alkaline medium (1M KOH).
• The data can be extracted from the repository (Mendeley) and re-plotted for further analysis or general comparison with specific characterizations (XRD deconvolution, XPS, CV and EIS).

## 1 Data Description

**Fig. 1:**
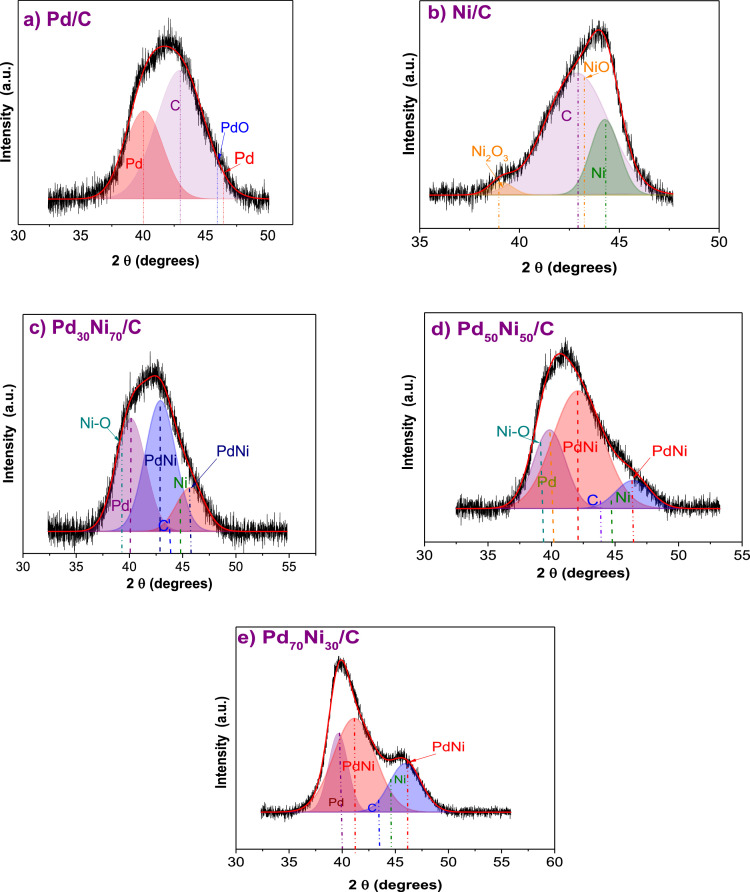
Deconvolution of X-ray diffraction patterns using the Gaussian function of a) Pd, b) Ni c) Pd_30_Ni_70_, d) Pd_50_Ni_50_ and e) Pd_70_Ni_30_.

**Fig. 2:**
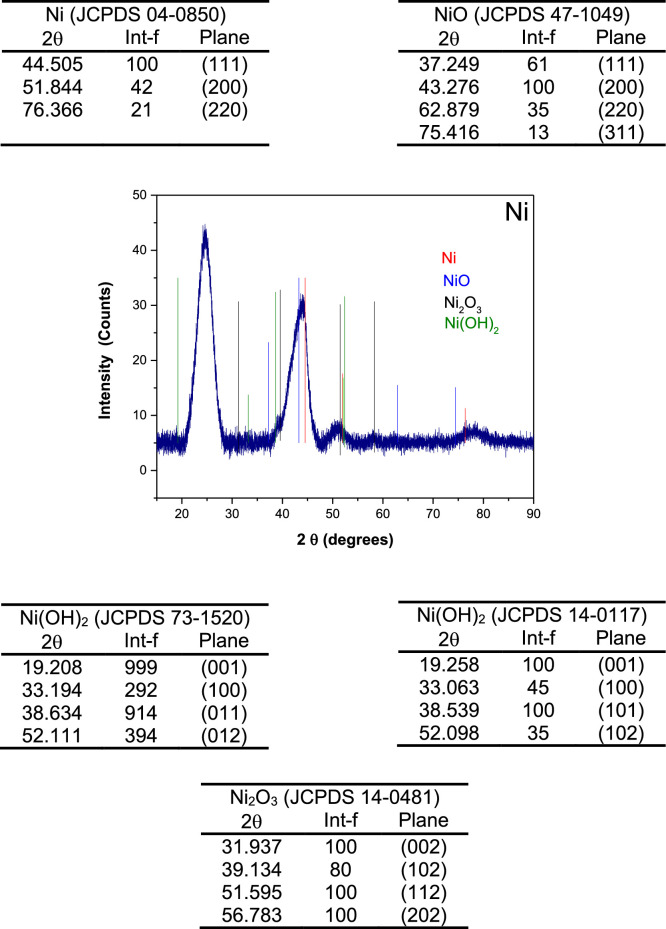
XRD patterns of Ni with their oxides and hydroxides compounds that were considered during deconvolution process.

**Fig. 3:**
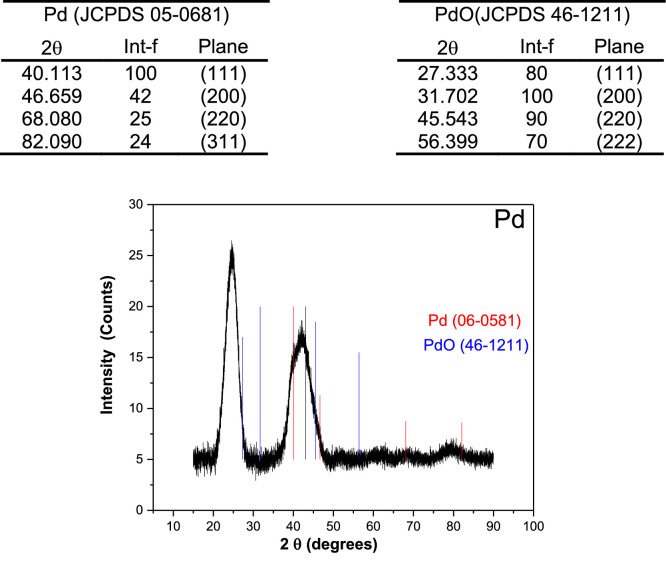
XRD patterns of Pd catalysts with its oxides compounds that were considered during deconvolution process.

The data set of the deconvolution XRD to separate the signal of carbon from Pd_x_Ni_1-x_ bimetallic materials and used to determinate in a better way the crystallite size is presented in Fig. 1 a-e. Figs. 2 and 3 show the intensity planes for Ni and Pd with their oxides/hydroxides that were considered during the deconvolution process. The addition of the Ni caused a reduction in the particle size.

**Table 1:** Chemical analysis of mono- and bi-metallic catalysis from XPS measurements.

Element	BE (eV)	FWHM (eV)	Peak Area (cts)	Area ratio (%)
**Pd**				

**Pd3d**				
Pd(II)	342.75	1.35	150.2	13.6
Pd(0)	341.7	1.17	258.7	23.5
Pd(II)	337.83	2.22	263.9	24
Pd(0)	336.44	1.28	428.1	38.9
**O1s**				
O-Pd	523.36	2.05	2271.5	100

**Pd_30_Ni_70_**				

**Ni2p**				
sat	882.79	4.0	140.26	2.54
Ni(II)	879.59	5.73	827.65	14.96
Ni(0)	873.60	4.36	919.54	16.62
sat	863.73	5.58	720.28	13.02
Ni(II)	861.29	3.07	606.33	10.96
Ni(0)	855.92	4.65	2317.74	41.90
O1s				
O-OH-Ni, OH-Ni	534.06	2.25	457.1	21.2
O-Pd, O-Ni, O-C	532.56	2.51	1695.2	78.8
**Pd3d**				
Pd(II)	341.46	1.56	133.00	20.82
Pd(0)	340.68	2.00	116.28	18.21
Pd(II)	336.74	2.00	175.37	27.48
Pd(0)	335.62	1.53	214.04	33.49

**Pd_50_Ni_50_**				

**Ni2p**				
sat	880.38	4.84	393.32	13.32
Ni(II)	877.59	1.89	45.82	1.55
Ni(0)	873.62	4.20	507.31	17.18
sat	863.31	4.70	355.41	12.04
Ni(II)	861.13	3.03	323.51	10.95
Ni(0)	855.85	4.57	1327.76	44.96
**O1s**				
O-OH-Ni, OH-Ni	534.49	2.25	566	27.5
O-Pd, O-Ni, O-C	532.68	2.27	1488.6	72.5
**Pd3d**				
Pd(II)	342.44	1.14	124	18.6
Pd(0)	341.81	0.93	147.4	22.1
Pd(II)	337.33	1.27	147	22
Pd(0)	336.52	1.09	249.8	37.4

**Pd_70_Ni_30_**				

**Ni2p**				
sat	881.15	5.59	284.63	12.22
Ni(II)	878.14	3.21	113.30	4.87
Ni(0)	873.52	4.30	355.45	15.26
sat	865.68	7.72	235.03	10.09
Ni(II)	861.69	4.32	390.43	16.77
Ni(0)	855.72	5.10	949.72	40.79
**O1s**				
O-OH-Ni, OH-Ni	535.97	1.81	197.2	9.8
O-Pd, O-Ni, O-C	532.94	3.2	1812.4	90.2
**Pd3d**				
Pd(II)	341.97	0.93	109.6	9.0
Pd(0)	340.86	1.07	403.9	33.2
Pd(II)	336.49	1.01	206.3	16.9
Pd(0)	335.56	0.92	498.5	40.9
**Ni**				

**Ni2p**				
sat	879.96	5.72	681.27	17.49
Ni(II)	873.60	3.97	648.68	16.65
Ni(0)	870.46	1.25	83.42	2.14
sat	861.55	5.32	880.50	22.61
Ni(II)	855.87	3.50	1251.47	32.13
Ni(0)	853.27	1.58	349.64	8.98
**O1s**				
O-OH-Ni, OH-Ni	534.31	2.42	580.1	29.5
O-Ni, O-C	532.53	2.07	1385.6	70.5

**Fig. 4:**
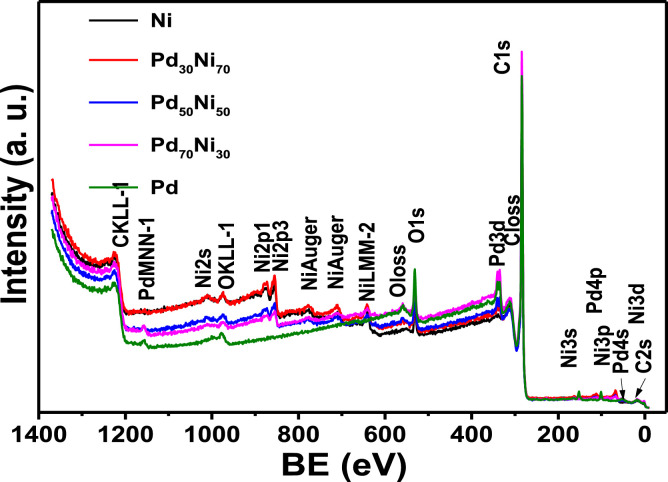
Low resolution X-ray photoelectron spectroscopy spectra of the samples used for the electrochemical performance of HER electrocatalysts in alkaline media; Pd, Ni, Pt_30_Ni_70_, Pt_50_Ni_50_ and Pt_70_Ni_30._

Fig. 4 displays XPS survey spectra recorded for the surface of as-obtain mono- and bi-metallic materials. From these spectra, the range of binding energy for each metal composition in the high resolution was determined. Also, it was used to evaluate changes in the electronic properties during alloy formation (Table 1).

**Fig. 5:**
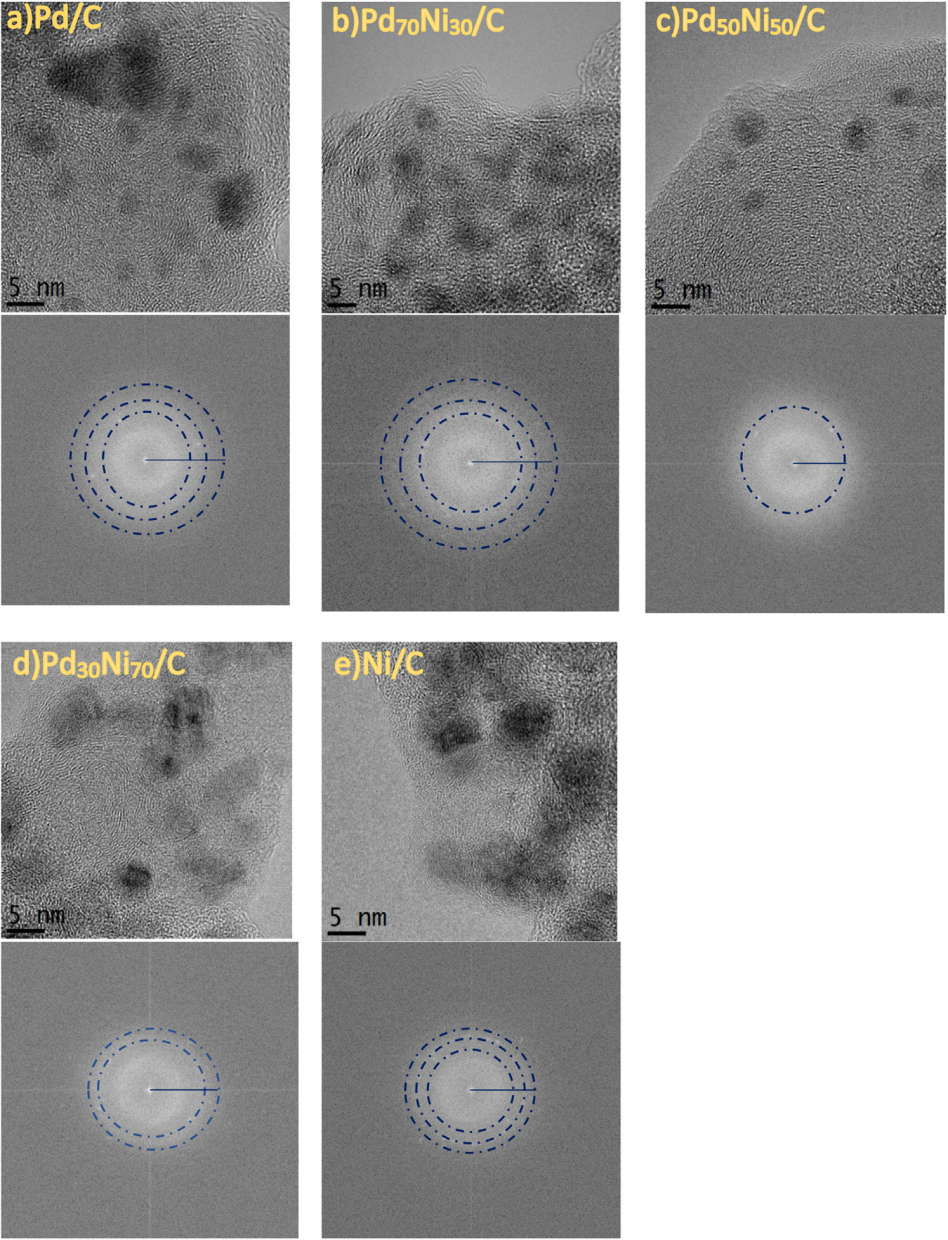
HRTEM morphologies of the mono and bimetallic nanocatalysts obtained from ligand displacement method and its corresponding SAED patterns after annealing process at 300°C; a) Pd/C, b) Pt_70_Ni_30_/C, c) Pt_50_Ni_50_/C, e) Pt_30_Ni_70_/C and d) Ni/C electrode materials.

**Fig. 6:**
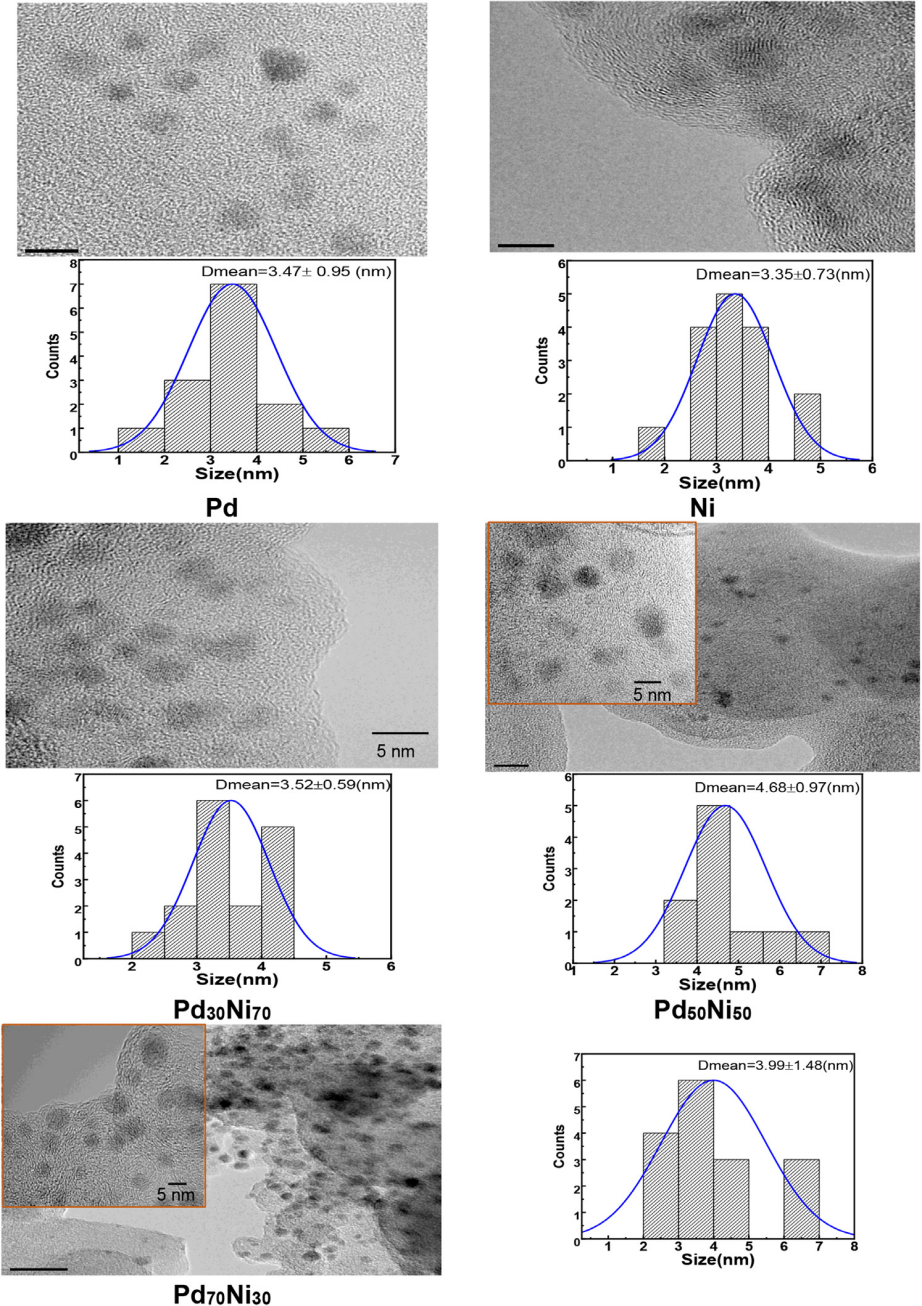
HRTEM morphologies of the mono and bimetallic nanocatalysts obtained from ligand displacement method and its corresponding average particle size after annealing process at 300°C

Figs. 5 and 6 show microstructural aspects with its corresponding selected area of electron diffraction (SAED) and histograms of particle size distribution in the as-prepared electrode materials. In these figures, the ring of SAED patterns matched with PdNi alloy plane. It is also seen that average particle size of all samples were lower than 6 nm suggesting an adequate stabilization with the proposed method.

**Fig. 7:**
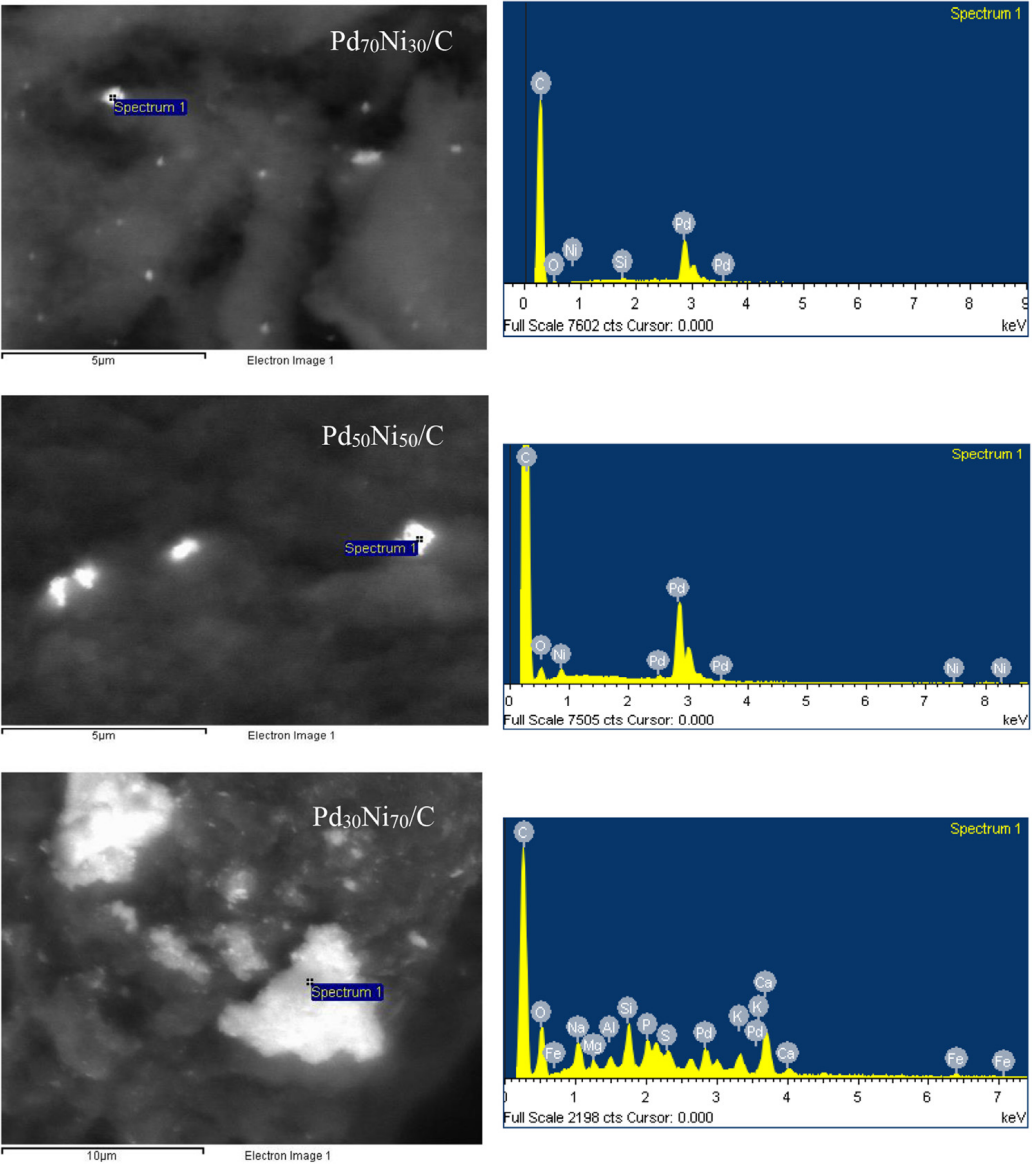
SEM/EDS analysis of the bimetallic Pd_30_Ni_70_/C, Pd_50_Ni_50_/C and Pd_70_Ni_30_/C catalysts.

Fig. 7 shows the compositional analysis of bimetallic nanoparticles in different regions of the samples.

**Fig. 8:**
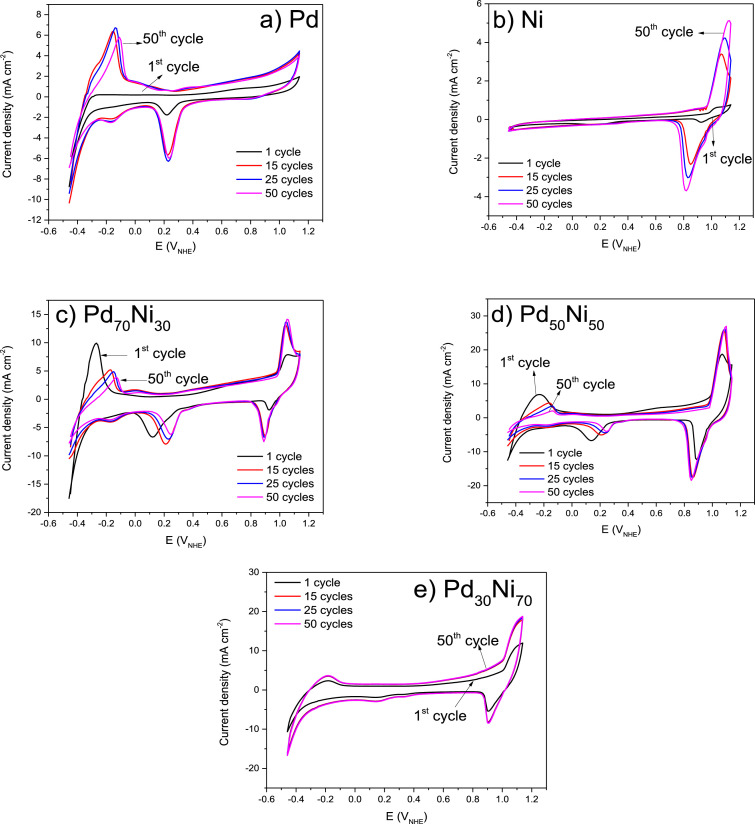
Cyclic voltammograms of a) Pd, b) Ni, c) Pd_70_Ni_30_, d) Pd_50_Ni_50_ and e) Pd_30_Ni_70_, evaluated in 1 M KOH

Fig. 8 a-e shows the CV diagrams realized for the stabilization of the electrode materials in N_2_ purge KOH (1 M) electrolyte during 50^th^ potential cycles. The synergistic effect by the Ni addition is observed in the voltammograms. The high current densities were observed for Pd_30_Ni_70._

**Fig. 9:**
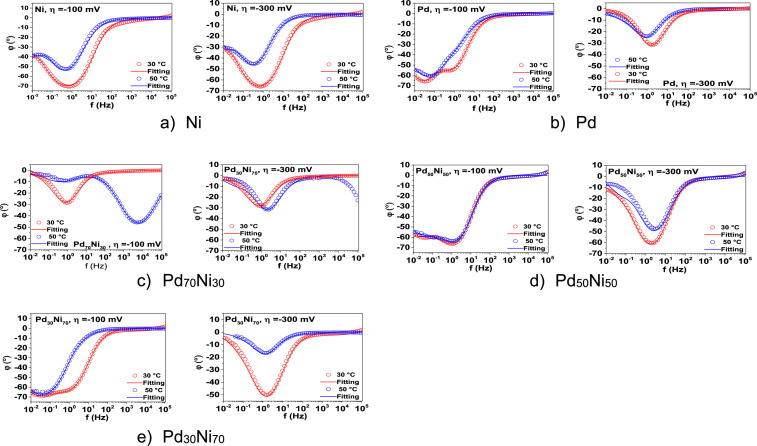
Phase angle representation with its corresponding fitting of the (a) Ni/C, (b) Pd/C, (c) Pd_70_Ni_30_/C (d) Pd_50_Ni_50_/C (e) Pd_30_Ni_70_/C electrode materials in a 1M KOH solution. The measurements were realized at two different overpotential (-100 and -300 mV) from the HER region and two different temperatures.

In Fig. 9, it is seen the complement EIS measurement in a representation of Bode diagrams (phase angle) at different overpotentials (-100 and 300 mV) and cell temperatures (30 and 50°C), confirming the previous performance of the electrodes for HER in alkaline medium.

## 2 Experimental Design, Materials and Methods

### 2.1 Preparation of electrode materials

The catalysts were prepared by ligands displacement of organometallic compounds [2]. Monometallic Pd, Ni and bimetallic Pd:Ni nanoparticles nominal weight percent ratios 70:30, 50:50 and 30:70 were synthesized from Pd(dba)_2_ and Ni(cod)_2_ precursors [3], [4], [5] and HDA as stabilizer. The mono- and bi-metallic catalysts were in-situ supported on Vulcan carbon (XC 72R) and the nominal metallic load was adjusted to 10 wt.%. The Pd nanoparticles were synthesized by mixing 0.5475 mmol of Pd(dba)_2_ and 0.5145 g of vulcan carbon, 2 equiv. of HDA (1.034 mmol) and 50 mL of A-THF in a Fisher-Porter bottle under nitrogen atmosphere. The procedure was replicate for obtaining bimetallic nanostructures, although in this case, the amount of Pd(dba)_2_ and Ni(cod)_2_ precursors was adjusted to obtain the following compositions Pd_x_Ni_1-x_ (x = 0, 30, 50, 70 and 100 wt.%) maintaining a theoretical ratio of Vulcan carbon: metallic content of 90:10. After synthesis, the as-synthesized materials were heated at 300°C under argon atmosphere for 2 h and after cooling for 24 h under the reducing atmosphere. Further details of the experimental procedure were presented in the reference [1].

### 2.2 Microstructure and chemical characterization

Pd base catalyst were structurally characterized by X-ray diffraction (XRD) using a Bruker D8 advanced diffractometer equipped with a Lynxeye detector and Cu Kα radiation (λ=1.5405 Å), which was operated at 35 kV, 25 mA and a scan rate of 0.016 min^−1^. The evaluated range was between 20° to 100° (2θ). Structural and morphological aspects of the as-prepared electrocalysts were also evaluated by high resolution transmission electron microscopy (HR-TEM) using a JEOL EM-ARM200CF apparatus, with an applied voltage of 200 kV.

The chemical composition of the films was characterized by X-ray photoelectron spectroscopy (XPS) spectra using a PHI-548 equipment. The spectra were acquired between 0-1400 eV at low resolutions using a source Al (Kα = 1486.6 eV), a step of 1.0 eV and pass energy of 100 eV with an operating pressure of 1 × 10^−8^ Torr. Collected data were analyzed with a Shirley background subtraction, performed with a Gaussian-Lorentzian profile.

### 2.3 Electrochemical characterization

The electrochemical characterization was performed with a classical three-electrode system, where an Hg/HgSO_4_ electrode was used as a reference, a platinum mesh was used as a counter electrode and the working electrode was a glassy carbon, GC (5 mm in diam.). The working electrode was prepared with 1.5 mg of as-prepared powders that were mixed with 10 µL of Nafion® + 185 µL of ethanol and sonicated for 20 min to form a uniform suspension (ink). The electrochemical measurements were carried out using an AUTOLAB (Metrohm, 50404) pontentiostat/galvanostat electrochemical workstation. The potentials were converted to the scale of normal hydrogen electrode (NHE). The scans were realized within the potential range from -1.2 V to 0.700 V vs Hg/HgSO_4_, but the results are presented vs NHE in a 1 M KOH solution saturated with an inert atmosphere (N_2_, Infra 99.999% purity) and 50 cycles were used to stabilize the electrode materials at a scan rate of 10 mV•s^−1^.

## Ethics Statement

Not applicable

## CRediT Author Statement

**J.J. de la Cruz-Cruz:** Conceptualization, Methodology, Validation; **M.A. Domínguez-Crespo:** Conceptualization, Visualization, Validation, Data curation; **E. Ramírez-Meneses:** Methodology, Visualization, Data curation; **A.M. Torres-Huerta:** Writing – original draft, Resources, Visualization; **S.B. Brachetti-Sibaja:** Formal analysis, Review & editing; **A.E. Rodríguez-Salazar:** Writing, Project administration, Review & editing; **E. Pastor:** Supervision, Writing – review & editing; **L.E. González-Sánchez:** Investigation, Validation.

## Declaration of Competing Interest

The authors declare that they have no known competing financial interests or personal relationships that could have appeared to influence the work reported in this paper.

## Data Availability

Data supporting the in situ synthesis by organometallic method of vulcan supported PdNi nanostructures for hydrogen evolution reaction in alkaline solution (Original data) (Mendeley Data).
